# Effect of ERAS-Based Rapid Bladder Irrigation on TURP and HoLEP in the Treatment of Benign Prostatic Hyperplasia

**DOI:** 10.12669/pjms.41.3.9751

**Published:** 2025-03

**Authors:** Shuhui An, Haoxuan Yang, Qingsong Meng

**Affiliations:** 1 Shuhui An, Department of Urology, The Second Hospital of Hebei Medical University, Shijiazhuang 050000, Hebei, China; 2 Haoxuan Yang, Department of Urology, The Second Hospital of Hebei Medical University, Shijiazhuang 050000, Hebei, China; 3 Qingsong Meng, Department of Urology, The Second Hospital of Hebei Medical University, Shijiazhuang 050000, Hebei, China

**Keywords:** Transurethral Resection of the Prostate (TURP), Holmium Laser Enucleation of the Prostate (HoLEP), Enhanced Recovery after Surgery (ERAS), Rapid Bladder Irrigation

## Abstract

**Objective:**

To explore the positive effect of rapid bladder irrigation in postoperative BPH patients by collecting and analyzing the efficacy and complications of TURP and HoLEP treatments for patients with benign prostatic hyperplasia (BPH) under ERAS care.

**Methods:**

A retrospective analysis was conducted on 197 BPH patients undergoing surgical treatment at the Second Hospital of Hebei Medical University from April 2022 to February 2024. All patients received rapid bladder irrigation under ERAS care, which involves flushing the bladder with a larger volume of saline solution in a shorter time postoperatively compared to conventional bladder irrigation care. Afterward, clinical data and postoperative irrigation indicators for each group were collected and analyzed.

**Results:**

Patients in the T1 group were observed with larger prostate volume, longer operation time, and more intraoperative blood loss than those in the T2 group, but with shorter duration of indwelling urinary catheters, shorter irrigation time, and shorter hospital stays (P<0.05). Meanwhile, the H1 group saw lower mean irrigation volume and shorter irrigation time than the H2 group. Differences were found between the T1 and H1 groups in terms of mean irrigation volume and irrigation time (P<0.05), Additionally, patients in the T2 group had lower prostate volume than those in the H2 group, with differences in mean irrigation volume and irrigation time (P<0.05).

**Conclusion:**

ERAS concept complements rapid bladder irrigation, which shortens the time to self-care recovery and discharge, thereby facilitating faster postoperative recovery for BPH patients.

## INTRODUCTION

As a common chronic urinary disease that causes lower urinary tract symptoms (LUTS) in men, benign prostatic hyperplasia (BPH) has an incidence of above 50% in men older than 50^1^ while affecting almost all elderly men, significantly impacting their urinary function and quality of life.^2^,^3^ In clinical practice, routine treatments for BPH include transurethral resection of the prostate (TURP), holmium laser enucleation of the prostate (HoLEP), and photoselective vaporization of the prostate (PVP). However, regardless of surgical procedures, comprehensive perioperative care and postoperative bladder irrigation are essential for patient recovery.^4^

Based on evidence-based medical practice, the concept of Enhanced Recovery after Surgery (ERAS) involves optimizing the clinical pathway of perioperative care through collaboration among surgical, anesthesia, nursing, and nutrition departments to alleviate perioperative stress responses, mitigate postoperative complications, shorten hospital stays, and promote patient recovery. This optimized clinical pathway spans throughout the entire diagnostic and treatment process from pre-admission to post-discharge, with a focus on the patient-centered approach.

Regardless of the surgical methods used for prostate resection, postoperative bladder irrigation is necessary, and the effects of different irrigation methods vary depending on the surgical methods performed.^5^,^6^ Specifically, rapid bladder irrigation involves flushing a larger volume of saline solution into the bladder in a shorter time postoperatively compared to conventional irrigation methods. In this study, ERAS was combined with rapid bladder irrigation to identify the optimal postoperative care for BPH patients, so as to reduce the perioperative psychological and physiological distress for patients.

## METHODS

A retrospective analysis was conducted on the clinical data of 197 BPH patients admitted to the Second Hospital of Hebei Medical University from April 2022 to February 2024. The patients were divided into the TURP group (n=127) and the HoLEP group (n=70) according to different surgical methods performed. The patients were divided into the T1 and T2 groups for TURP postoperative rapid and conventional bladder irrigation care, respectively, and the H1 and H2 groups for HoLEP postoperative rapid and conventional bladder irrigation care, respectively. And all patients were diagnosed as BPH into the bladder by color ultrasound before surgery.

### Ethical Approval:

The study was approved by the Institutional Ethics Committee of the Second Hospital of Hebei Medical University (No.: 2024-R048; date: February 23, 2024), and written informed consent was obtained from all participants.

### Inclusion criteria:

- Patients with no other underlying diseases affecting the study results.
- Preoperative education was provided by urologists in our hospital, with patients signing the informed consent for surgery and physicians at the same level performing the surgery.
- Patients underwent further preoperative examinations in our hospital 1-3 days before surgery.
- Patients with no contraindications to surgery upon examinations.
- Patients actively underwent postoperative recovery following the doctor’s advice after surgery, complemented with corresponding care.

### Exclusion criteria:

- Patients with suspected prostate cancer or other prostate diseases.
- Patients who changed the surgical method or didn’t undergo TURP or HoLEP during the operation.
- Patients with other combined urinary system diseases, such as a urethral stricture.
- Patients who rejected follow-ups and investigations.
- Patients with other systemic complications occurring during operation that affected their discharge, such as concurrent respiratory tract infection.

The TURP group consisted of 127 patients with a mean age of (69.83 ± 7.83) years, prostate volume of (51.35 ± 16.26) mL, and mean operation time of (81.80 ± 40.83) min. There were 70 patients in the HoLEP group, with a mean age of (69.94 ± 5.94) years, prostate volume of (54.80 ± 19.23) mL, and mean operation time of (78.26 ± 34.88) min. No significant differences were observed in the general profile between the two groups (Table-I) (P > 0.05).

**Table-I:** Comparison of General Profile of Patients with 2 Surgical Methods.

Surgical method	Age/year	Prostate volume/ml	Operation time/min	Blood loss/ml
BPH	69.83±7.83	51.35±16.26	84.51±46.82	48.33±38.82
HoLEP	69.94±5.94	54.80±19.23	77.39±35.89	39.59±26.46
P	0.91	0.05	0.89	0.055

### Perioperative Management:

All patients received ERAS care (see the Discussion section for specific measures). Additionally, after returning to the ward postoperatively, all patients were connected to 0.9% sodium chloride injection at 35°C (3L). Rapid bladder irrigation was performed at a rate of 160-240 drops/min for the first eight hours. Afterward, the color of the patient’s urine was monitored, with the irrigation fluid rate adjusted according to the color changes. The irrigation was stopped once the fluid became completely clear and colorless. In contrast, the conventional bladder irrigation was performed at a rate of 80-120 drops/min, with an interval of at least 40 hours within 1-3 days postoperatively.

Meanwhile, the color of the irrigation fluid was monitored, with continuous irrigation changing to slow or intermittent irrigation if the color changed from dark to light, and the irrigation was stopped if there were no symptoms of bleeding or urinary obstruction. All patients received routine antibiotic prophylaxis to prevent infection and were instructed to clean the urethral meatus daily. Before removing the urinary catheter, patients were advised to drink plenty of water, intermittently clamp the catheter, and if the patient’s condition allowed, the catheter was removed as early as possible. Three to five days after the operation, the catheter clipping was performed within 24 hours before the removal of the catheter to train the bladder function.

### Observation Indicators:

The patients were divided into the TURP and HoLEP groups, which were further divided into the T1, T2, H1, and H2 subgroups based on whether rapid bladder irrigation was performed. Specifically, the T1 and T2 groups received rapid and conventional bladder irrigation care after TURP, respectively, and the H1 and H2 groups received rapid and conventional bladder irrigation care after HoLEP, respectively. Meanwhile, an analysis was conducted on the differences in terms of operation time, intraoperative blood loss, postoperative hospital stays, and hospitalization expenses to compare the different prostate resection procedures. Additionally, the postoperative irrigation fluid volume, irrigation time, time to the first defecation, off-bed time, duration of indwelling urinary catheters, and other indicators were statistically analyzed to evaluate the speed of postoperative recovery for patients in the two groups.

### Statistical Analysis:

SPSS 26.0 software was used for the statistical analysis, and P<0.05 was considered statistically significant. The general profile of patients and irrigation indicators were expressed as mean ± standard deviation. Control variables were compared between the four groups using pairwise comparisons. The t-test was utilized for data following the normal distribution, with the Mann-Whitney U-test used for data not following the normal distribution.

## RESULTS

All patients underwent successful surgeries without serious complications and were reexamined in our outpatient department two months postoperatively, indicating excellent recovery and no need for a second surgery. The statistical analysis suggested no significant differences in age among the four groups, (Table-II).

**Table-II:** Comparison of Efficacy of 2 Irrigation Methods for Different Surgical Methods.

General Profile						Irrigation indicators			
Surgical method	Case	Age/year	Prostate volume/ml	Operation time/min	Intraoperative blood loss/ml	Duration of indwelling urinary catheter /d	Mean irrigation volume/ml/h	Irrigation time/h	Hospital stays/d
T1	65	69.34±6.92	56.57±16.59	94.32±44.71	58.00±45.89	7.05±5.23	30.57±9.25	33.95±20.89	1.89±1.13
T2	62	70.35±7.75	45.87±14.05	68.66±31.67	40.16±27.59	9.05±4.58	30.90±6.70	52.42±35.56	3.35±2.21
H1	35	68.63±6.05	56.57±14.74	77.77±36.17	41.71±25.20	5.03±3.49	21.14±4.09	22.66±10.47	1.66±0.91
H2	35	71.26±5.51	53.03±11.91	78.74±34.06	32.57±19.45	5.54±4.19	24.97±6.54	41.51±21.96	1.89±0.96
P (T1, T2)	—	0.373	<0.01	<0.01	0.019	0.002	0.331	<0.01	<0.01
P (H1, H2)	—	0.122	0.259	0.869	0.139	0.924	0.008	<0.01	0.321
P (T1, H1)	—	0.893	0.899	0.17	0.104	0.119	<0.01	<0.01	0.324
P (T2, H2)	—	0.38	0.007	0.141	0.337	<0.01	<0.01	0.086	<0.01

Despite no significant differences in the mean irrigation volume between the T2 group and the T1 group (P > 0.05), the total amount of irrigation fluid used in the T1 group was lower than in the T2 group due to the longer irrigation time in the latter. No significant differences were observed in age, prostate volume, operation time, and intraoperative blood loss between patients in the H1 and H2 groups (P > 0.05), and there was a difference between mean irrigation volume and irrigation time (P < 0.05). However, there were no significant differences in hospital stays and the duration of indwelling urinary catheters between the two groups (P > 0.05).

There was no significant difference in age, prostate volume, operation time, and intraoperative blood loss between the T1 and H1 groups (P > 0.05), but significant differences were observed in mean irrigation volume and irrigation time (P < 0.05). However, no significant difference existed in hospital stays and the duration of indwelling urinary catheters between the two groups (P > 0.05).

Comparison between the H2 and T2 groups showed no significant differences in age, operation time, and intraoperative blood loss (P > 0.05), while the T2 group had a lower prostate volume (45.87 ± 14.05ml) than the H2 group (53.03 ± 11.91 ml), with significant differences in mean irrigation volume and irrigation time (P < 0.05).

There may be some bias in the data of the T2 group due to the lower prostate volume, less operation time, and less intraoperative blood loss, while conventional bladder irrigation care was a preferred option because of the uncertainty about the role of rapid bladder irrigation in TURP care, resulting in differences in the general profile of patients. In spite of this, the T2 group didn’t predominate in various postoperative irrigation indicators, which also indirectly demonstrated the advantages of rapid bladder irrigation care.

## DISCUSSION

HoLEP surgery is characterized by less blood loss and shorter operation time compared to traditional TURP surgery.^7^ Despite no significant difference between the two surgeries observed in this study, patients undergoing HoLEP surgery had larger prostate volumes, but relatively shorter operation times and less blood loss compared to those undergoing TURP surgery, proving that Holmium Laser Enucleation of the Prostate (HoLEP) definitely show certain but not significant advantages over Transurethral Resection of the Prostate (TURP). Due to the steeper learning curve and more expensive equipment associated with HoLEP surgery, TURP surgery is still one of the preferred surgical procedures in clinical practice.^8^ The comparison between the T1 and H1 groups T1 suggested that the length of hospital stays and the duration of the indwelling urinary catheter were similar under the dual care strategy of Enhanced Recovery After Surgery (ERAS) and rapid bladder irrigation, demonstrating that ERAS care and rapid bladder irrigation can reduce the probability of postoperative complications and the difference in postoperative recovery speed between the two procedures. Despite being initially applied for rapid recovery in gastrointestinal surgeries^9^, the concept of ERAS has rapidly spread to various types of surgeries beyond gastrointestinal surgeries since it was introduced, including various surgeries for treating prostate enlargement. A set of ERAS care procedures has been developed by our hospital for reference by peers in the field.

Currently, prostatectomy is the most common treatment for BPH, and proper care is particularly important for accelerating the recovery of patients and improving their prognosis. Regardless of surgical procedures, postoperative bladder irrigation is an important part of postoperative care.^10^,^11^ Due to the large resection area and uneven resection surface during prostatectomy, some hidden bleeding spots may be difficult to identify, and blood stasis may accumulate in the bladder postoperatively, forming blood clots. In severe cases, it can block the ureter or urethra. If blood clots cannot be expelled, a bladder fistula or even a bladder incision may be needed to remove the blood clots, aggravating the injury of patients.^12^

An ERAS team consisting of urologists, anesthesiologists, and nurses was established by the Second Hospital of Hebei Medical University. Based on the characteristics of BPH, a tailored accelerated recovery nursing strategy was developed for patients, complemented with corresponding nursing measures.

### Preoperative care:

- Urologists provided patients with information about the disease and surgery, emphasizing the effectiveness and safety of the surgery. Nurses highlighted the ERAS recovery process and precautions to keep patients emotionally stable and increase their compliance with the surgery to actively cooperate with treatment.^13^
- Physicians and nurses developed targeted nursing guidance based on patients’ condition, such as guiding patients with lower limb disabilities to defecate in a supine position before surgery. Patients were also instructed on Kegel exercises to prevent stress urinary incontinence.^14^ Additionally, tailored psychological care was provided to patients based on their different mental states to encourage and support them and boost their confidence.
- Nutritional risk assessment was performed upon admission using the Nutritional Risk Screening 2002 (NRS-2002) scale. Patients with a total score of ≥3 were given healthy dietary advice and preoperative nutritional support.^15^
- Patients fasted for 8 h before surgery, consuming easily digestible, high-nutrient, high-fiber foods like egg custard and high-fiber biscuits. For patients without a history of diabetes, 200 ml of 12.5% carbohydrate beverage (e.g., Powerade) could be orally administered two hours or above preoperatively to maintain fluid balance and internal stability while avoiding abnormal blood sugar fluctuations. Water intake was completely restricted to two hours before surgery.^16^
- Patients were assisted in cleaning the perineum preoperatively, with skin preparation performed on the day of surgery to reduce the risk of skin infections. Intraoperative care: Prophylactic antibiotics, such as cefazolin sodium, were given 30 min before surgery. Patients were assisted to adjust to lithotomy position, along with strengthened ECG monitoring and warming interventions. In addition, anesthesiologists closely monitored the vital signs of patients and promptly reported any abnormalities to the surgeon for necessary intervention, ensuring the volume of intraoperative fluid replacement within 1,500 ml to prevent heart failure due to excessive fluid.

### Postoperative care:

- Prophylactic antibiotics were continued postoperatively, but the total duration of postoperative medication should not exceed 48 hour. Additionally, Flurbiprofen axetil was applied for pain relief, with phloroglucinol or anisodamine used to relieve spasms.^17^
- Patients could drink a small amount of water once awake. If no discomfort occurred, they could take a small amount of liquid diet and gradually transition to a full liquid diet and semi-liquid diet. Patients were advised to chew gum to stimulate gastrointestinal function recovery.
- Patients were instructed to engage in bedside exercises, turn over regularly, and customize the off-bed time according to their individual condition. Generally, patients were advised to move for 1-2 hours on the first day after surgery, increasing to 4-6 hours daily after catheter removal. Psychological counseling was also provided to reduce patient anxiety.
- Continuous bladder irrigation was performed using 36°C saline. During the first eight hour, the bladder irrigation rate should be 160-240 drops/min. Afterward, the irrigation time and rate were reasonably adjusted based on the color of the irrigation fluid, and the urinary catheter was removed as soon as the patient’s condition allowed. Respiratory care should be provided throughout the entire perioperative period^18^. In view of this, the risk of respiratory system complications during hospitalization and anesthesia could be minimized through the above measures.^19^

Currently, numerous reports are available on postoperative care for BPH,^6^,^20^ and experts have also concluded corresponding expert consensus. However, there is still a lack of specific guidance on bladder irrigation, with the majority of studies relying on the color of the irrigation fluid to adjust the postoperative bladder irrigation flow rate, which is actually inaccurate. In this study, rapid bladder irrigation has been found to accelerate patient recovery under the condition that the irrigation rate was used as a variable and other care modes were consistent, demonstrating the feasibility and scientific basis of rapid bladder irrigation. Meanwhile, rapid bladder irrigation also reduces the probability of postoperative complications of BPH.

However, since the incidence of postoperative complications is related to factors such as the size of the resected prostate tissue, intraoperative conditions, and the baseline status of patients, variables cannot be satisfactorily controlled. Therefore, the incidence of complications was not included in the statistical analysis of the study. However, from the clinical feedback, it appears that patients undergoing rapid bladder irrigation experience milder postoperative symptoms compared to those undergoing regular bladder irrigation.

### Limitations:

It includes a small number of samples and no long-term follow-up was conducted. In view of this, more samples should be included and follow-up time should be increased in future studies to further validate the findings of this study.

## CONCLUSIONS

In conclusion, rapid bladder irrigation can improve irrigation indicators and accelerate patient recovery. The statistically significant differences in postoperative clinical indicators between the two groups (P <0.05) indicate that postoperative rapid bladder irrigation is of great significance to different surgical procedures. The concept of ERAS complements rapid bladder irrigation, which shortens the time to self-care and discharge for patients. Therefore, it is hoped that these improvements can help BPH patients recover faster and more effectively, thereby benefiting more patients.

### Authors’ Contributions:

**SA:** Literature search, performed the study**,** and drafted the manuscript.

**HY** and **QM:** Concept, Design**,** Performed the statistical analysis. Critical review.

All authors have read, approved the final manuscript. They are also responsible and accountable for the accuracy or integrity of the work.
